# Neutrophil-to-lymphocyte ratio, platelet-to-lymphocyte ratio, and monocyte-to-lymphocyte ratio in depressed patients with suicidal behavior: A systematic review

**DOI:** 10.1192/j.eurpsy.2023.18

**Published:** 2023-04-16

**Authors:** A Velasco, A Lengvenyte, J Rodriguez-Revuelta, L Jimenez-Treviño, P Courtet, MP Garcia-Portilla, J Bobes, PA Sáiz

**Affiliations:** 1 Department of Psychiatry, University of Oviedo, Oviedo, Spain; 2 Centro de Investigación Biomédica en Red de Salud Mental (CIBERSAM), Instituto de Salud Carlos III, Madrid, Spain; 3 Instituto de Investigación Sanitaria del Principado de Asturias (ISPA), Oviedo, Spain; 4 Instituto de Neurociencias del Principado de Asturias (INEUROPA), Oviedo, Spain; 5 Department of Emergency Psychiatry and Acute Care, CHU de Montpellier, Montpellier, France; 6 IGF, University of Montpellier, CNRS, INSERM, Montpellier, France; 7 Psychiatric Clinic, Institute of Clinical Medicine, Faculty of Medicine, Vilnius University, Vilnius, Lithuania; 8 Mental Health Services of the Principality of Asturias (SESPA), Oviedo, Spain

**Keywords:** Depression, monocyte/lymphocyte ratio, neutrophil/lymphocyte ratio, platelet/lymphocyte ratio, suicidal behavior

## Abstract

**Background:**

Inflammatory biomarkers are reportedly increased in depressed patients. Several studies have been conducted using neutrophil/lymphocyte ratio (NLR), platelet/lymphocyte ratio (PLR), and monocyte/lymphocyte ratio (MLR). The objective of this systematic review was to study the relationship between these peripheral biomarkers and suicidality in depressed patients with/without suicidal behavior, including suicide attempts and ideation, and healthy controls.

**Methods:**

We searched the following relevant terms in the PubMed, Web of Science, and Scopus databases published in the last 5 years. We assessed the methodological quality of included studies using the Oxford criteria and reviewed the evidence following PRISMA guidelines.

**Results:**

Eleven studies were retained for the data synthesis, with a total sample of 1,701 participants, of which the majority (819) were patients with depression and suicidal behavior, 494 were depressed patients without suicidal behavior, and only 388 were healthy participants. Our results reinforce the idea that NLR could be an attractive, convenient, and cost-effective trait marker of suicidal vulnerability in patients with major depressive disorder (MDD).

**Conclusion:**

Future large-scale replication studies are needed to examine the apparently understudied role of PLR and MLR in depressed patients in greater depth.

## Introduction

Suicidal behavior (SB) is a serious public health concern. More than 700,000 people die by suicide every year, representing one death every 40 s on average [1]. Factors contributing to increased risk of SBs are diverse and complex, but epidemiological studies indicate that the vast majority of attempted and completed suicides occur in people with mental disorders with mood disorders being the most frequently associated with SB [2, 3].

Understanding the pathophysiology of suicide is still a long-term goal. The evidence increasingly indicates a possible role of the immune-inflammatory response in the development and maintenance of depression and SB [4]. Inflammation has been associated with an increased risk of SBs above and beyond the risk associated with depression [5]. Neuroinflammatory processes are a pathophysiological mechanism that is essential for understanding SB in depressed patients. To explain the role of the immune system in the pathophysiology of suicide, a comprehensive model has been proposed. In this model, sleep disturbances, stress, childhood abuse, and infections induce dysregulation of the hypothalamic–pituitary–adrenal (HPA) axis that is associated with a chronic low-grade inflammatory state and increased risk of SBs [6]. It has therefore been suggested that inflammatory biomarkers are potentially useful in predicting and monitoring suicide risk in patients with depression [7].

Neutrophil-to-lymphocyte ratio (NLR), platelet-to-lymphocyte ratio (PLR), and monocyte-to-lymphocyte ratio (MLR) indexes are convenient and cost-effective blood indicators of inflammatory status [8].

NLR is the most studied hematological parameter [9]. Neutrophils are the first defense cells of the innate immune system, representing an active nonspecific inflammatory mediator of phagocytosis and apoptosis functions [10], and lymphocytes represent the regulatory or protective component of the immune system [11–13]. NLR is the ratio between two different immune pathways reflecting the intensity of chronic stress. It may be more informative and perhaps less changed by unknown factors. PLR index is related to stress. The presence of stress activates the sympathetic nervous system, increases platelets, and induces endothelial permeability. When this permeability occurs, neutrophils and macrophages appear, generating peripheral inflammation [14]. Some studies suggest that PLR could be a better predictor than NLR for determining the severity of inflammation [15, 16]. An elevated level of MLR is associated with an overexpression of immunological genes that increases the production of cytokines related to monocytes and, as a consequence, activates microglia in the brain, causing neuroinflammation [14].

These indexes have been suggested as new indicators of low-grade inflammation and have been used as systemic inflammation prognostic scores in diseases such as cancer, coronary heart disease, and pancreatitis [17] and are also being investigated in neuropsychiatric disorders such as Alzheimer’s disease, schizophrenia, bipolar disorder, and major depressive disorder (MDD) [11, 18]. Recently, a meta-analysis [18] reported that inflammatory activation occurs in mood disorders and that NLR and PLR may be useful to detect this activation. NLR has been found to increase in depressed patients compared with healthy controls (HCs) [15, 19] and in depressed patients with SB. Moreover, studies have suggested that NLR may be a significant predictor of SB in MDD [4, 20] and could be more elevated in patients with recent suicide attempt (SA) [21]. In parallel, increased PLR levels have also been associated with the diagnosis and severity of depression [15, 22]. Finally, MLR was significantly higher in adolescents with SA than in HC [23].

However, potential mechanisms underlying inflammatory processes in depression and SB have yet to be fully elucidated. Biomarkers would provide more personalized methods for their assessment and treatment and would help to enhance our understanding of suicidal pathophysiology and improve prevention [24]. No previous reviews have examined NLR, PLR, and MLR in depressed patients with/without SA and suicidal ideation (SI) and HC. Therefore, we aimed to explore if there are significant differences in NLR, PLR, and MLR in (i) depressed patients with or without a lifetime history of SA; (ii) depressed patients with a lifetime history of SA vs HCs; and (iii) depressed patients with SI before and after treatment.

## Materials and Methods

A systematic literature search was conducted according to the Preferred Reporting Items for Systematic Reviews and Meta-Analyses (PRISMA) Statement [25]. The protocol was registered with the International Prospective Register of Systematic Reviews (PROSPERO) (CRD42022361238).

### Search criteria

We systematically searched PubMed, Web of Science, and Scopus databases in September 2022. A single search strategy has been used for each of the databases:

Studies of neutrophil-to-lymphocyte ratio were systematically searched using the terms “(NLR OR neutrophil-to-lymphocyte ratio OR neutrophil-to-lymphocyte index OR neutrophil-to-lymphocyte rate OR neutrophil to lymphocyte ratio OR neutrophil to lymphocyte index OR neutrophil to lymphocyte rate OR neutrophil–lymphocyte ratio OR neutrophil lymphocyte index OR neutrophil lymphocyte rate OR neutrophil/lymphocyte ratio OR neutrophil/lymphocyte index OR neutrophil/lymphocyte rate) AND (depressive disorder OR depressive disorders OR depression OR mood disorder OR mood disorders OR major depression) AND (suicide OR suicidal behavior OR suicide attempt OR suicidal thoughts OR self-mutilation).”

In the same way, studies of platelet-to-lymphocyte ratio were systematically searched using the terms “(PLR OR platelet-to-lymphocyte ratio OR platelet-to-lymphocyte index OR platelet-to-lymphocyte rate OR platelet to lymphocyte ratio OR platelet to lymphocyte index OR platelet to lymphocyte rate OR platelet lymphocyte ratio OR platelet lymphocyte index OR platelet lymphocyte rate OR platelet/lymphocyte ratio OR platelet/lymphocyte index OR platelet/lymphocyte rate) AND (depressive disorder OR depressive disorders OR depression OR mood disorder OR mood disorders OR major depression) AND (suicide OR suicidal behavior OR suicidal attempt OR suicidal thoughts OR self-mutilation).”

Finally, studies of monocyte-to-lymphocyte ratio were systematically searched using the terms “(MLR OR monocyte-to-lymphocyte ratio OR monocyte-to-lymphocyte index OR monocyte-to-lymphocyte rate OR monocyte to lymphocyte ratio OR monocyte to lymphocyte index OR monocyte to lymphocyte rate OR monocyte lymphocyte ratio OR monocyte lymphocyte index OR monocyte lymphocyte rate OR monocyte/lymphocyte ratio OR monocyte/lymphocyte index OR monocyte/lymphocyte rate) AND (depressive disorder OR depressive disorders OR depression OR mood disorder OR mood disorders OR major depression) AND (suicide OR suicidal behavior OR suicidal attempt OR suicidal thoughts OR self-mutilation).”

We reviewed titles and abstracts to select potentially relevant papers. After this screening process, we reviewed the full texts and checked the references in the included studies, meta-analyses, and systematic reviews to identify additional studies. Some data were extracted from previous meta-analyses and systematic reviews.

### Eligibility criteria

Case–control studies and cross-sectional data from longitudinal studies that compared NLR, PLR, and/or MLR indexes among depressed patients with SB, depressed patients without SB, and HCs were included, based on the following criteria: (i) patients with MDD according to standardized diagnostic criteria; (ii) measurement of NLR, PLR, and/or MLR in young people and adults; (iii) patients with current SI and history of SB. Only articles in English published in the last 5 years were included. Conference and meeting abstracts, meta-analyses, reviews, and pilot studies were excluded (see Supplementary Table S1).

### Data extraction and assessment of methodological quality

Data were extracted by two independent authors (AV and PAS) and verified by the other two (JR and LJ). Extracted data included author, year of publication, country, diagnosis, study population, sample size, age, ratios of females, depression and suicide scales, type of outcome (SI/SA), and type and quality of the study. Results were ordered according to indexes (Table 1). The methodological quality of the included studies was assessed using the Oxford criteria [33]. Only medium- and high-quality papers were included in the final review. Any disagreements between reviewers were resolved by discussion and consensus.

**Table 1.** Main characteristics and results of articles included in the review.

Author, date Country	Diagnosis	Total sample mean age (SD) sex (% females)	Suicidal behavior mean age (SD) sex (% females)	Control patients mean age (SD) sex (% females)	Healthy controls mean age (SD) sex (% females)	Depression and suicidal ideation scales	SI/SA group	Type and quality of study	Outcome
Amitai et al. [26] Israel	Mostly depression + anxiety	*N* = 91 13.9 (2.42) years 56 (62%) females	*N* = 22 15 (65%) females	*N* = 69	Not available	K-SADS-PL CDRS-R C-SSRS	SA	Case–control 2c	NLR and PLR at baseline were higher in SA group than in nSA group
									NLR and PLR correlate with SI
									NLR was a better predictor of SB than PLR
									After 8 weeks of fluoxetine treatment, NLR and PLR indexes were higher but not significantly
Adhikari et al. [27] India	MDD	*N* = 50 39.27 (10.15) years 26 (52%) females	Not available	Not available	Not available	MADRS SIS-MAP	SI	Longitudinal 2c	Baseline: males and females do not differ in any blood parameters, except for a higher total white blood cell count in males
									After pharmacotherapy (12 weeks), in females only, neutrophils were increased, lymphocytes decreased and consequently, NLR was increased in response to antidepressant therapy In males, NLR was associated with decreased depressive symptoms and suicide risk, and higher CRP
Ekinci and Ekinci, [20] Turkey	MDD	*N* = 189 43 (9.98) years 97 (69.7%) females	*N* = 37 43 (14.15) years 22 (77.3%) females	*N* = 102 41.88 (11.49) years 75 (64.3%) females	*N* = 50 44.12 (4.23) 37 (74%) females	HDRS	SA	Case–control 3b	NLR was higher in patients with SA than in nSA and HC subjects. NLR was a predictor of recent SA in MDD patients
									PLR was not significant
Velasco et al. [4] Spain	MDD	*N* = 538 43.87 (14.36) years 370 (68.8%) females	*N* = 402 41.20 (13.78) years 289 (71.9%) females	*N* = 136 51.77 (13.10) years 81 (59.6%) females	Not available	HDRS	SA	Cross-sectional 2c	NLR was higher in MDD patients with SA vs MDD patients without SA. NLR was a biological marker of SB with a cut-off value of 1.30, 75% sensitivity, and 35% specificity
									PLR was higher in MDD patients with SA vs MDD patients without SA
									MLR did not show any differences between MDD patients with or without a history of SA
Demirkol et al. [8] Turkey	MDD	*N* = 74 Age not available 50 (67.5%) females	Not available	Not available	Not available	HDRS BSSI	SI	Longitudinal 2c	AD + BLT showed significant improvement over AD monotherapy
									HDRS scores decreased significantly 1 day after treatment and continued for 2 weeks after
									SI decreased significantly after treatment
									Neutrophils were decreased, lymphocytes were increased and consequently, NLR was decreased significantly in all patients after treatment
									PLR index did not differ before or after treatment
Grudet et al. [28] Sweden	MDD	*N* = 102 38.6 (14.4) years 60 (59%) females	*N* = 17 42.1 (14.3) years 9 (53%) females	*N* = 31 37.7 (15.2) years 18 (58%) females	*N* = 54 37.9 (13.9) 33 (61%) females	HDRS Items SI (HDRS) > 4	SI	Case–control 3b	NLR did not show differences between MDD vs HC or between MDD with SI vs without SI
									Lower levels of vitamin D were more associated with a pro-inflammatory status in MDD patients than HC, especially in MDD patients with SI
Puangsri and Ninla-Aesong, [9] Thailand	MDD	*N* = 193	*N* = 81 MDD SI n = 38 MDD SA n = 43	MDD nSI *n* = 56	*N* = 56 20 (1.1) years 36 (64.29%) females	PHQ-9 DASS-21 8Q	SA SI	Case- control 3b	NLR was significantly higher in all MDD patients vs HC
									PLR was significantly higher in all MDD patients vs HC PLR was higher in MDD nSI vs HC
									MLR in MDD SA was significantly higher than HC and in MDD nSI MLR tended to be higher in the MDD SI group
Martínez-Botía, Velasco and Rolle et al. [29] Spain	MDD	*N* = 172 50.08 (11.19) years 86 (50%) females	*N* = 48 50.94 (9.71) years 30 (62.5%) females	*N* = 31 54.35 (11.61) years 16 (51.6%) females	*N* = 93 48.22 (11.44) years 40 (43.0%) females	HDRS	SA	Case–control 3b	NLR was not statistically significant where found between groups
									PLR was not statistically significant where found between groups
									MLR was more significantly decreased with SA than MDD nSA and HC
Yagci and Avci [30] Turkey	Mostly depression + anxiety	*N* = 91 32.53 (10.39) years 57 (62.6%) females	*N* = 46 33 (71.7%) females	Not available	*N* = 45 24 (53.3.%) females	BDI BAI BSSI	SA	Case–control 3b	NLR was higher in SA than in HC NLR positively correlated between BDI, BAI, and BSSI. However, this correlation was not statistically significant
Önen et al. [31] Turkey	MDD	*N* = 148; *N* = 58 MDD patients 13.97 (1.98) years 44 (75.9%) females	*N* = 15	*N* = 43	*N* = 90 14.29 (1.67) years 60 (66.7%) females	CDI	SB (not specified if SA or SI)	Case–control 3b	NLR did not show any differences between groups
									PLR was higher in the MDD group than in HC. PLR was a biological marker of MDD in children and adolescents with a cut-off value of 112.5, 70% sensitivity, and 65% specificity
Gundogdu Meydaneri and Meydaneri [32] Turkey	MDD	*N* = 53 33 (12) years 40 (75%) females	*N* = 27 32 (13) years 20 (74%) females	*N* = 26 35 (11) years 20 (77%) females	Not available	None	SA	Cross-sectional 2c	NLR tended to be higher in the SA group. However, this difference was not statically significant
									PLR tended to be higher in the SA group. However, this difference was not statically significant

Abbreviations: AD, antidepressant; BAI, beck anxiety inventory; BDI, beck depression inventory; BLT, bright light therapy; BSSI, beck scale for suicide ideation; CDI, children’s depression inventory; CDRS-R, children’s depression rating scale- revised; CRP, C-reactive protein; C-SSRS, Columbia-suicide severity rating scale; DASS-21, depression anxiety stress scale; HC, healthy control; HDRS, Hamilton depression rating scale; K-SADS-PL, kiddie-schedule for affective disorders & schizophrenia – present and lifetime version; MADRS, Montgomery–Asberg depression rating scale; MDD, major depressive disorder; MLR, monocyte/lymphocyte ratio; NLR, neutrophil/lymphocyte ratio; nSA, non-suicide attempt; nSI, non-suicidal ideation; PHQ-9, patient health questionnaire; PLR, platelet/lymphocyte ratio; SA, suicide attempt; SB, suicidal behavior; SI, suicidal ideation; SD, standard deviation; SIS-MAP, scale for impact of suicidality management and assessment and planning of care; 8Q, 8 questionnaire (Thai-version of a suicidality module of Mini International Neuropsychiatric Interview).

## Results

### Study selection and characteristics

**Figure 1.**
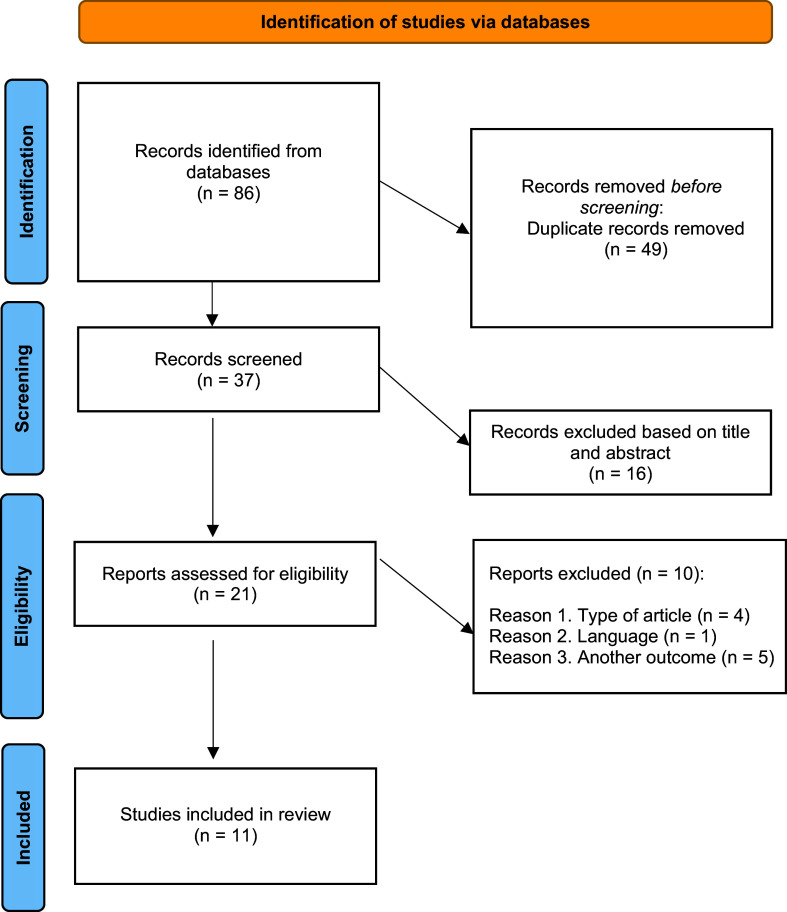
PRISMA 2020 flow diagram for new systematic reviews that included database searches.

A total of 86 studies were identified from electronic databases and, after removing duplicates, there were 37 single records to be screened. After reading titles and abstracts, we identified 21 full-text articles to be assessed for eligibility but excluded 10 studies after the full text was read (the inclusion and exclusion process is depicted in Figure 1). Of those, 11 studies met the inclusion and quality criteria and were selected for this review.

A total of 10 papers were rejected for the following reasons: (i) type of article (review, letter, meeting abstract): two articles were meeting abstracts and two articles were letters to the editor; (ii) two articles did not include a study of SB, two articles did not specify depressed patients, and one study included parameters of peripheral inflammation other than NLR, PLR, and MLR; and (iii) one article was published in the Turkish language (see Supplementary Table S1).

All included studies were published between August 2017 and September 2022. Five studies were conducted in Turkey [8, 20, 30–32], one in India [27], two in Spain [4, 29], one in Sweden [28], one in Israel [26], and one in Thailand [9].

The 11 records included in the review yielded a total sample of 1,701 participants, of which 819 were patients with MDD and SB (including current SI and lifetime SA), 494 were control patients (MDD without SB), and 388 were HCs.

We included data from (i) six studies of NLR, PLR, and/or MLR in depressed patients with or without SB [4, 20, 26, 28, 29, 32]; (ii) six studies of NLR, PLR, and/or MLR in depressed patients with SB vs HC [9, 20, 28–31]; (iii) two studies of NLR in depressed patients with SI before (with or without monotherapy 4 weeks prior) and after pharmacotherapy (2 to 12 weeks after treatment), one of which also explored PLR [8, 27], respectively (Table 1).

### Patients with major depressive disorder with or without suicidal behavior

In three studies with patients with moderate-to-severe depression, NLR was reported to be higher in suicide attempters compared with depressed patients without a history of SA [4, 20, 26]. NLR could be potentially used as a biomarker to predict recent and past SA [4]. However, these results were not confirmed in other studies. First, in two studies there was a (nonsignificant) tendency toward an increase in NLR in patients with a history of SA vs without any [29, 32]. One study examining the association between current SI and NLR in patients with MDD found no differences between patients with and without current SI in NLR [28] (Table 2).

**Table 2.** Neutrophil-to-lymphocyte ratio, platelet-to-lymphocyte ratio, and monocyte-to-lymphocyte ratio in major depressive disorder with suicidal behavior versus non-suicidal behavior patients.

Author, date	Type of SB	MDD with SB	MDD without SB	
NLR		Mean (SD)	Mean (SD)	*p*-value
Velasco et al. [4]	Recent SA Past SA	2.37 (2.36) 2.22 (2.17)	1.68 (0.80)	**0.001**
Amitai et al. [26]	SA	2.16 (0.78)	1.64 (0.96)	**0.019**
Ekinci and Ekinci [20]	Recent SA	2.840 (0.162)	1.858 (0.98)	**0.001**
Gundogdu Meydaneri and Meydaneri [32]	SA	2.04 (0.89)	1.85 (0.81)	0.054
Martínez-Botía et al. [29]	SA	2.17 (1.66)	1.68 (0.57)	0.291
Grudet et al. [28]	SI	2.3 (1.0)	2.2 (0.8)	0.81

PLR		Mean (SD)	Mean (SD)	*p*-value
Velasco et al. [4]	Recent SA Past SA	128.20 (61.65) 127.76 (58.91)	109.97 (38.75)	**0.024**
Amitai et al. [26]	SA	159.31 (53.98)	133.56 (58.18)	**0.044**
Ekinci and Ekinci [20]	Recent SA	141.4 (83.25)	128.11 (48.76)	0.248
Gundogdu Meydaneri and Meydaneri [32]	SA	120.81 (39.34)	118.55 (42.39)	0.73
Martínez-Botía et al. [29]	SA	122.39 (64.51)	109.30 (28.50)	0.416

MLR		Mean (SD)	Mean (SD)	*p*-value
Velasco et al. [4]	Recent SA Past SA	0.28 (0.16) 0.27 (0.15)	0.25 (0.10)	0.573
Martínez-Botía et al. [29]	SA	0.26 (0.12)	0.25 (0.08)	0.851

Abbreviations: MDD, major depressive disorder; MLR, monocyte/lymphocyte ratio; NLR, neutrophil/lymphocyte ratio; PLR, platelet/lymphocyte ratio; SA, suicide attempt; SB, suicidal behavior; SI, suicidal ideation; SD, standard deviation.

Regarding the PLR index in patients with MDD, two studies reported PLR to be higher in patients with a history of SA vs those without any [4, 26]. Conversely, this difference was not observed in three other studies [20, 29, 32] (Table 2).

Finally, only two studies explored the MLR index in relation to SB in MDD, with no reported statistically significant differences between depressed patients with and without SB [4, 29] (Table 2).

### Patients with major depressive disorder and suicidal behavior vs healthy controls

In three studies, NLR was reported to be higher in depressed patients with a history of SA vs HC [9, 20, 30]. However, three other studies reported no statistically significant differences [28, 29, 31] (Table 3).

**Table 3.** Neutrophil-to-lymphocyte ratio, platelet-to-lymphocyte ratio, and monocyte-to-lymphocyte ratio in major depressive disorder with suicidal behavior versus healthy controls.

Author, date	MDD with SB	Healthy controls	
NLR	Mean (SD)	Mean (SD)	*p*-value
Ekinci and Ekinci [20]	2.840 (0.162)	1.81 (0.33)	**0.001**
Puangsri and Ninla-Aesong [9]	2.01 (0.07)	1.49 (0.04)	**0.001**
Yagci and Avci [30]	2.77 (1.6)	2.05 (0.59)	**0.009**
Martínez-Botía et al. [29]	1.97 (1.35)	1.87 (0.80)	0.510
Grudet et al. [28]	2.3 (1.0)	2.3 (1.2)	0.81
Önen et al. [31]	1.94 (1.11)	1.65 (0.66)	0.780

PLR	Mean (SD)	Mean (SD)	*p*-value
Puangsri and Ninla-Aesong [9]	123.18 (3.35)	105.80 (3.11)	**0.024**
Önen et al. [31]	133.95 (41.65)	114.44 (40.53)	**0.005**
Ekinci and Ekinci [20]	141.4 (83.25)	134.3 (61.4)	0.248
Martínez-Botía et al. [29]	117.26 (53.49)	117.99 (54.77)	0.952

MLR	Mean (SD)	Mean (SD)	*p*-value
Puangsri and Ninla-Aesong [9]	0.21 (0.02)	0.15 (0.01)	**0.027**
Martínez-Botía et al. [29]	0.26 (0.11)	0.30 (0.13)	**0.017**

Abbreviations: MDD, major depressive disorder; MLR, monocyte/lymphocyte ratio; NLR, neutrophil/lymphocyte ratio; PLR, platelet/lymphocyte ratio; SA, suicide attempt; SB, suicidal behavior; SI, suicidal ideation; SD, standard deviation.

Regarding the PLR index, two studies reported that PLR was higher in depressed patients (including SA, SI, and nSI) vs HC [23, 30]. Conversely, in two studies, this difference was not observed [20, 29] (Table 3).

MLR was investigated in only two out of six studies, with inconsistent results. MLR was reported to be higher in depressed patients with SA vs HC [9]. However, MLR was reported to be decreased in depressed patients with SB vs HC [29] (Table 3).

### Depressed patients with suicidal ideation before and after treatment for depression

There were two studies that evaluated NLR before and after antidepressant treatment. First, Demirkol et al. (2019) studied depressed patients (*n* = 74) with monotherapy 4 weeks before treatment and found a decrease in NLR during and after treatment with antidepressant therapy (AD) and bright light therapy (BLT), with a greater decrease compared with AD monotherapy [NLR mean (SD) = 2.31(1.05) pretreatment vs 2.25 (0.96) after treatment vs 1.9 (0.9) 2 weeks after treatment; *p* < 0.001]. In addition, HDRS scores and SI were also significantly decreased after treatment [HDRS mean (SD) = 20.69 (4.21) pretreatment vs 16.69 (5.96) after treatment vs 15.14 (5.33) 2 weeks after treatment; *p* < 0.001; and SI median (Q1–Q3): 5 [3–10] pretreatment vs 5 [1–9] after treatment vs 3.5 (0–7) 2 weeks after treatment; *p* < 0.001] [8]. However, in a sample of 50 depressed patients without antidepressant treatment the previous month, Adhikari et al. [27] found a significant increase in NLR after 12 weeks of antidepressant treatment only in females [NLR mean in females (SD) = 2.55 (0.87) pretreatment vs 2.85 (0.89) 12 weeks after treatment; p < 0.001].

Regarding PLR, only one study examined PLR levels in depressed patients with SI before and after treatment and concluded there was no significant change in PLR during AD treatment [PLR mean (SD) = 118.61 (39.01) pretreatment vs 118.16 (40.57) after treatment vs 117.96 (40.82) 2 weeks after treatment; *p* = 0.985] [8].

None of the studies included MLR.

## Discussion

SB is a leading cause of death and disability worldwide [34]. Detecting and identifying potential biomarkers of peripheral inflammation in SB has the potential to provide the knowledge needed to understand the pathophysiology of SB, develop personalized therapies, and improve prevention. To date, this is the first review that has examined NLR, PLR, and MLR in depressed patients with and without SA and current SI versus HCs.

NLR, PLR, and MLR indexes emerge as relatively stable biomarkers of systemic inflammation [35], which, in turn, is cost-effective and easily accessible. Perhaps, for this reason, most of the studies included in this systematic review were conducted in low-income countries, which seem to be interested in this option given the potential value for clinical application.

According to our review, in depressed patients, NLR was higher in patients with a history of SA, suggesting that, if confirmed in larger studies, it could be a biomarker of suicidal vulnerability in these patients. Although this result was not observed in all studies, in the majority there was a tendency for increased NLR. To the best of our knowledge, only one study that examined the differences in patients with and without SI did not find this association [28]. However, it has been previously suggested that SA and SI are different phenomena with different explanations and predictors [36], and it seems that the relationship between the increase in NLR and suicidality occurs only in SA and not in SI [28]. In addition, NLR was higher in depressed patients with SA vs HC. However, these differences were not observed in depressed patients with SI vs HC. Mounting evidence indicates that activation of the immune-inflammatory response is linked to the development and maintenance of depression and SB [4, 7, 18].

Some studies suggest that PLR could be a better predictor than NLR for determining the severity of inflammation [15, 16]. Our review shows inconsistent results regarding PLR in patients with MDD (including SB). However, when compared with HC, the difference in the PLR index is observed more clearly in depressed patients. This phenomenon might be explained by the fact that platelets are one of the first cells to start an inflammatory cascade (cytokines, chemokines, the serotonin pathway), and patients with depression had a loss of equilibrium in hematopoietic production, resulting in an imbalance or distress in modulation [29].

The present review found no evidence for the link between MLR and SB in depressed patients, in contrast to some studies than showed MLR was higher in the manic episodes of bipolar disorder compared with euthymic states [37]. However, despite not being related to SB, high MLR in young people appears to be associated with self-harm when compared with young people without this behavior [14].

Finally, discrepant, and limited results were found regarding SI changes and inflammatory indexes following antidepressant treatment. These results may indicate that: (i) not all depressed patients show changes in the inflammatory response [27]; (ii) the mechanisms underlying the relationship between inflammation and suicide are still unclear [38]: and (iii) it has not been determined whether inflammation is a causative factor or a consequence of depression [11]. However, we also need to keep in mind that inflammatory response is influenced by multiple factors, such as body mass index, use of tobacco and other psychoactive substances, duration and severity of illness, resistance to antidepressant treatment, other psychiatric comorbidities, unbalanced diet, lack of exercise, and stress or traumatic life events in childhood or adulthood [27, 29, 39].

## Limitations

First, our results are mostly based on cross-sectional studies, and a causal relationship between NLR and SI and SB in patients with MDD cannot be inferred. Second, other inflammatory parameters were not assessed in the review, precluding us from concluding whether increased NLR is an independent biomarker or is related to other immune and inflammatory changes in depressed patients. Third, the studies included in the systematic review are very heterogeneous (i.e., sample characteristics, small sample sizes, recruitment, and assessment of depression and suicidality), and therefore results remain preliminary and cannot be generalized to any specific population. Finally, due to the small number and small scale of studies included, we cannot exclude publication and reporting bias in those studies, possibly biasing the results of the systematic review.

## Conclusion

In conclusion, the present review found preliminary evidence for an association between NLR and SB in patients with MDD. Our results reinforce the idea that neuroinflammatory processes may be important in the pathophysiology of SB in depressed patients. NLR could be an attractive, convenient, and cost-effective trait marker of suicidal vulnerability in patients with MDD. Future large-scale replication studies are needed to confirm the observed associations and to examine the apparently understudied role of PLR and MLR in depressed patients in greater depth.

## Supporting information

Velasco et al. supplementary material Velasco et al. supplementary material

## Supplementary material

The supplementary material for this article can be found at http://doi.org/10.1192/j.eurpsy.2023.18.

## References

[1] World Health Organization. Suicide worldwide in 2019: global health estimates. World Health Organization. Licence: CC BY-NC-SA 3.0 IGO, https://apps.who.int/iris/rest/bitstreams/1350975/retrieve; (accessed on 21 April 2021).

[2] Bachmann S. Epidemiology of suicide and the psychiatric perspective. Int J Environ Res Public Health. 2018;15(7):1425. doi:10.3390/ijerph15071425. 29986446 PMC6068947

[3] Dong M, Wang SB, Wang F, Zhang L, Ungvari GS, Ng CH, et al. Suicide-related behaviours in schizophrenia in China: a comprehensive meta-analysis. Epidemiol Psychiatr Sci. 2019;28(3):290. doi:10.1017/S2045796017000476. 28944747 PMC6998905

[4] Velasco Á, Rodríguez-Revuelta J, Olié E, Abad I, Fernández-Peláez A, Cazals A, et al. Neutrophil-to-lymphocyte ratio: a potential new peripheral biomarker of suicidal behavior. Eur Psychiatry. 2020;63(1):e14. doi:10.1192/j.eurpsy.2019.20. 32093807 PMC7315873

[5] Bergmans RS, Kelly KM, Mezuk B. Inflammation as a unique marker of suicide ideation distinct from depression syndrome among U.S. adults. J Affect Disord. 2019;245:1052. doi:10.1016/j.jad.2018.11.046. 30699847 PMC6448785

[6] Vasupanrajit A, Jirakran K, Tunvirachaisakul C, Solmi M, Maes M. Inflammation and nitro-oxidative stress in current suicidal attempts and current suicidal ideation: a systematic review and meta-analysis. Mol Psychiatry. 2022;27(3):1350–61. doi:10.1038/s41380-021-01407-4. 34997194

[7] Courtet P, Giner L, Seneque M, Guillaume S, Olie E, Ducasse D. Neuroinflammation in suicide: toward a comprehensive model. World J Biol Psychiatry. 2016;17(8):564–86. doi:10.3109/15622975.2015.1054879. 26223957

[8] Demirkol ME, Namlı Z, Tamam L. Efficacy of light therapy on non-seasonal depression and inflammatory markers. Eur J Psychiatry. 2019;33(3):104–11. doi:10.1016/j.ejpsy.2019.03.002.

[9] Puangsri P, Ninla-aesong P. Potential usefulness of complete blood count parameters and inflammatory ratios as simple biomarkers of depression and suicide risk in drug-naive, adolescents with major depressive disorder: simple peripheral markers for inflammation in drug-naive, adolescents with major depressive disorder. Psychiatry Res. 2021;305:114216. doi:10.1016/j.psychres.2021.114216. 34571404

[10] Rungelrath V, Kobayashi SD, DeLeo FR. Neutrophils in innate immunity and systems biology-level approaches. Wiley Interdiscip Rev Syst Biol Med. 2020;12(1):e1458. doi:10.1002/wsbm.1458. 31218817 PMC6898734

[11] Özyurt G, Binici NC. Increased neutrophil-lymphocyte ratios in depressive adolescents is correlated with the severity of depression. Psychiatry Res. 2018;268:426–31. doi:10.1016/j.psychres.2018.08.007. 30130709

[12] Chaplin DD. Overview of the immune response. J Allergy Clin Immunol. 2010;125(2 Suppl 2):S3–23. doi:10.1016/j.jaci.2009.12.980. 20176265 PMC2923430

[13] Ivković M, Pantović-Stefanović M, Dunjić-Kostić B, Jurišić V, Lačković M, Totić-Poznanović S, et al. Neutrophil-to-lymphocyte ratio predicting suicide risk in euthymic patients with bipolar disorder: moderatory effect of family history. Compr Psychiatry. 2016;66:87–95. doi:10.1016/j.comppsych.2016.01.005. 26995241

[14] Zheng Q, Liu J, Ji YJ, Zhang Y, Chen XC, Liu BS. Elevated levels of monocyte-lymphocyte ratio and platelet-lymphocyte ratio in adolescents with non-suicidal self-injury. BMC Psychiatry. 2022;22(1):618. doi:10.1186/s12888-022-04260-z. 36123674 PMC9483869

[15] Kayhan F, Gündüz Ş, Ersoy SA, Kandeğer A, Annagür BB. Relationships of neutrophil–lymphocyte and platelet–lymphocyte ratios with the severity of major depression. Psychiatry Res. 2017;247:332–5. doi:10.1016/j.psychres.2016.11.016. 27978453

[16] Turkmen K, Erdur FM, Ozcicek F, Ozcicek A, Akbas EM, Ozbicer A, et al. Platelet-to-lymphocyte ratio better predicts inflammation than neutrophil-to-lymphocyte ratio in end-stage renal disease patients. Hemodial Int. 2013;17(3):391–6. doi:10.1111/hdi.12040. 23522328

[17] Isaac V, Wu CY, Huang CT, Baune BT, Tseng CL, McLachlan CS. Elevated neutrophil to lymphocyte ratio predicts mortality in medical inpatients with multiple chronic conditions. Medicine (Baltimore). 2016;95(23):e3832. doi:10.1097/MD.0000000000003832. 27281085 PMC4907663

[18] Mazza MG, Lucchi S, Tringali AGM, Rossetti A, Botti ER, Clerici M. Neutrophil/lymphocyte ratio and platelet/lymphocyte ratio in mood disorders: a meta-analysis. Prog Neuro-Psychopharmacol Biol Psychiatry. 2018;84:229–36. doi:10.1016/j.pnpbp.2018.03.012. 29535038

[19] Demir S, Atli A, Bulut M, İbiloğlu AO, Ggüneş M, Kaya MC, et al. Neutrophil-lymphocyte ratio in patients with major depressive disorder undergoing no pharmacological therapy. Neuropsychiatr Dis Treat. 2015;11:2253–8. doi:10.2147/NDT.S89470. 26347335 PMC4556257

[20] Ekinci O, Ekinci A. The connections among suicidal behavior, lipid profile and low-grade inflammation in patients with major depressive disorder: a specific relationship with the neutrophil-to-lymphocyte ratio. Nord J Psychiatry. 2017;71(8):574–80. doi:10.1080/08039488.2017.1363285. 28800269

[21] Orum MH, Kara MZ, Egilmez OB. Mean platelet volume and neutrophil to lymphocyte ratio as parameters to indicate the severity of suicide attempt. J Immunoass Immunochem. 2018;39(6):647–59. doi:10.1080/15321819.2018.1529682. 30311834

[22] Sunbul EA, Sunbul M, Yanartas O, Cengiz F, Bozbay M, Sari I, et al. Increased neutrophil/lymphocyte ratio in patients with depression is correlated with the severity of depression and cardiovascular risk factors. Psychiatry Investig. 2016;13(1):121–6. doi:10.4306/pi.2016.13.1.121. PMC4701675 26766954

[23] Ucuz İ, Kayhan Tetik B. Can suicide behavior and seasonality of suicide be predicted from inflammatory parameters in adolescents? Med Hypotheses. 2020;143:110061. doi:10.1016/j.mehy.2020.110061. 32650198

[24] Dickerson F, Adamos M, Katsafanas E, Khushalani S, Origoni A, Savage C, et al. The association between immune markers and recent suicide attempts in patients with serious mental illness: a pilot study. Psychiatry Res. 2017;255:8–12. doi:10.1016/j.psychres.2017.05.005. 28505469

[25] Page M, McKenzie JE, Bossuyt PM, Boutron I, Hoffmann TC, Mulrow CD et al. The PRISMA 2020 statement: an updated guideline for reporting systematic reviews. BMJ. 2021;372:n71. doi:10.1136/bmj.n71. 33782057 PMC8005924

[26] Amitai M, Kaffman S, Kroizer E, Lebow M, Magen I, Benaroya-Milshtein N, et al. Neutrophil to-lymphocyte and platelet-to-lymphocyte ratios as biomarkers for suicidal behavior in children and adolescents with depression or anxiety treated with selective serotonin reuptake inhibitors. Brain Behav Immun. 2022;104:31–8. doi:10.1016/j.bbi.2022.04.018. 35470013

[27] Adhikari A, Dikshit R, Karia S, Sonavane S, Shah N, De Sousa A. Neutrophil-lymphocyte ratio and c-reactive protein level in patients with major depressive disorder before and after pharmacotherapy. East Asian Arch Psychiatr. 2018;28(2):53–8. 29921741

[28] Grudet C, Wolkowitz OM, Mellon SH, Malm J, Reus VI, Brundin L, et al. Vitamin D and inflammation in major depressive disorder. J Affect Disord. 2020;267:33–41. doi:10.1016/j.jad.2020.01.168. 32063570 PMC10662683

[29] Martínez-Botía P, Velasco A, Rolle V, Jiménez-Trevino L, De la Fuente-Tomás L, Bernardo Á, et al. Sex-dependent grades of haematopoietic modulation in patients with major depressive episodes are associated with suicide attempts. Eur Neuropsychopharmacol. 2020;40:17–30. doi:10.1016/j.euroneuro.2020.06.006. 32600963

[30] Yagci I, Avci S. Biochemical predictors in presentations to the emergency department after a suicide attemp. Bratisl Med J. 2021;122(3):224–9. doi:10.4149/BLL_2021_012. 33618533

[31] Önen Ö, Erkuran HÖ, Bağ Ö, Abacıgil F. Blood count parameters as inflammation indicators in children and adolescents diagnosed with depressive disorder. Psychiatry Clin Psychopharmacol. 2021;31(4):425–33. doi:10.5152/pcp.2021.21137. 38765642 PMC11079696

[32] Gundogdu Meydaneri G, Meydaneri S. Can neutrophil lymphocyte ratio predict the likelihood of suicide in patients with major depression? Cureus. 2018;10(4):e2510. doi:10.7759/cureus.2510. 29930888 PMC6007446

[33] Howick J, Chalmers I, Glasziou P, Greenhalgh T, Heneghan C, Liberati A, et al. Explanation of the 2011 Oxford Centre for Evidence-Based Medicine (OCEBM) Levels of Evidence (Background Document). Oxford Centre for Evidence-Based Medicine, https://www.cebm.ox.ac.uk/resources/levels-of-evidence/explanation-of-the-2011-ocebm-levels-of-evidence; (accessed on 17 June 2020).

[34] Klonsky E, May A, Saffer B. Suicide attempts, and suicidal ideation. Annu Rev Clin Psychol. 2016;12:307–30. doi:10.1146/annurev-clinpsy-021815-093204. 26772209

[35] Mazza MG, De Lorenzo R, Conte C, Poletti S, Vai B, Bollettini I, et al. Anxiety and depression in COVID-19 survivors: role of inflammatory and clinical predictors. Brain Behav Immun. 2020;89:594–600. doi:10.1016/j.bbi.2020.07.037. 32738287 PMC7390748

[36] Galfalvy H, Haghighi F, Hodgkinson C, Goldman D, Oquendo MA, Burke A, et al. A genome-wide association study of suicidal behavior. Am J Med Genet B Neuropsychiatr Genet. 2015;168(7):557–63. doi:10.1002/ajmg.b.32330. 26079190

[37] Özdin S, Sarisoy G, Böke Ö. A comparison of the neutrophil-lymphocyte, platelet-lymphocyte and monocyte-lymphocyte ratios in schizophrenia and bipolar disorder patients - a retrospective file review. Nord J Psychiatry. 2017;71(7):509–12. doi:10.1080/08039488.2017.1340517. 28644753

[38] Russell AE, Mars B, Wen CP, Chang S Sen, Gunnell D. Evidence for an association between inflammatory markers and suicide: a cohort study based on 359,849 to 462,747 Taiwanese adults. J Affect Disord. 2021;281:967–71. doi:10.1016/j.jad.2020.10.047. 33250203

[39] Del Giudice M, Gangestad SW. Rethinking IL-6 and CRP: why they are more than inflammatory biomarkers, and why it matters. Brain Behav Immun. 2018;70:61–75. doi:10.1016/j.bbi.2018.02.013. 29499302

