# Supplementary material for: Neutrophil-to-lymphocyte ratio, platelet-to-lymphocyte ratio, and monocyte-to-lymphocyte ratio in depressed patients with suicidal behavior: A systematic review
Source: Eur Psychiatry. 2023 Apr 16;67(1):e76. doi: 10.1192/j.eurpsy.2023.18 (PMC11730062; doi:10.1192/j.eurpsy.2023.18)
Supplement: Velasco et al. supplementary material [file S0924933823000184sup001.doc]

Supplementary Table. Studies Excluded from the Review with reasons.

| Reference | Reason(s) for exclusion |
| --- | --- |
| Torrigiani S, Flamini W, Ivaldi T, Marazziti D. Relevance of neutrophil/lymphocyte ratio, platelet/lymphocyte ratio and monocyte/lymphocyte ratio in mood disorders: a literature review. [European Neuropsychopharmacology: The Journal of the European College of Neuropsychopharmacology](https://www.researchgate.net/journal/European-neuropsychopharmacology-the-journal-of-the-European-College-of-Neuropsychopharmacology-0924-977X) 40:S142; P248. | Type of article: Abstract to European College of Neuropsychopharmacology Congress. |
| Giynas Ayhan M, Dagistan AA, Sen Tanrikulu C, Yildiz Bozdogan S, Eren I.  Increased neutrophil/lymphocyte ratio in suicide attempters. [Anadolu Psikiyatri Derg](https://www.bibliomed.org/?jtt=1302-6631)isi 2019;20(3): 305-312. doi: [10.5455/apd.8099](https://dx.doi.org/10.5455/apd.8099). | The study has been published in the Turkish language and is not available in English. |
| Buoli M, Capuzzi E, Caldiroli A, Ceresa A, Esposito C, Posio C, Auxilia AM, Capellazzi M, Tagliabue I, Surace T, Legnani F, Cirella L, Di Paolo M, Nosari G, Zanelli Quarantini F, Clerici M, Colmegna F, Dakanalis, A.  Clinical and Biological Factors Are Associated with Treatment-Resistant Depression. Behavioral Science (Basel) 2022;12(2): 34. doi: 10.3390/bs12020034. | The study does not include suicidal behavior and evaluates other parameters of peripheral inflammation. |
| Kumar K, Srivastava S, Sharma B, Avasthi RK, Kotru M. Comparison Between inflammatory Biomarkers (High-Sensitivity C-Reactive Protein and Neutrophil-Lymphocyte Ratio) and Psychological Morbidity in Suicide Attempt Survivors Brought to Medicine Emergency. Cureus 2021;13(8):e17459. doi:10.7759/cureus.17459. eCollection 2021 Aug. | The study does not specify that it includes depressed patients. |
| Ucuz I, Kayhan Tetik B. Can suicide behavior and seasonality of suicide be predicted from inflammatory parameters in adolescents? Medical Hypotheses. 2020;143:110061. | The study does not specify that it includes depressed patients. |
| Orum, M. H. Relationship between complete blood count parameters and suicide: The role of neutrophil to lymphocyte ratio. European Journal of Psychiatry. [Vol. 35. Issue 4.](https://www.elsevier.es/en-revista-european-journal-psychiatry-431-sumario-vol-35-num-4-S0213616321X00043)  Pages 276-277 (October - December 2021). | Type of article: letter to the editor. |
| De Diego-Adeliño J, Ávila-Parcet A, Real J, Puigdemont D, Portella MJ, Fernández-Vidal A. Neutrophil-lymphocyte index correlates with severity and suicidal intentionality in suicide attempts. European Neuropsychopharmacology: Abstract of the ECNP Workshop early career scientist. S-435. | Type of article: Abstract to ECNP Congress |
| Özyurt G, Binici NC. Increased neutrophil-to-lymphocyte ratios in depressive adolescents is correlated with severity of depression. Psychiatry Research.2018;268:426-431. | The study does not assess suicidal behavior |
| Keaton SA, Madaj ZB, Heilman P, Smart L, Grit J, Gibbons R, Postolache TT, Roaten K, Achtyes ED, Brundin L. An inflammatory profile linked to increased suicide risk. Journal of Affective Disorders.2019;247:57-65. | The study includes other parameters of peripheral inflammation different from NLR, PLR and MLR. |
| Kara MZ, Orum MH, Egilmez OB. Relationship between immune cells and violent/nonviolent suicide attempts and controls: What about the lymphocyte-related ratios and neutrophil-related parameters? The Kaohsiung Journal of Medical Science.2018;35:315-316. | Type of article: Letter to the editor. |
